# A lack of *Wolbachia*-specific DNA in samples from apollo butterfly (*Parnassius apollo*, Lepidoptera: Papilionidae) individuals with deformed or reduced wings

**DOI:** 10.1007/s13353-015-0318-1

**Published:** 2015-09-30

**Authors:** Kinga Łukasiewicz, Marek Sanak, Grzegorz Węgrzyn

**Affiliations:** Division of Molecular Biology and Clinical Genetics, Department of Medicine, Jagiellonian University Medical College, Skawińska 8, 31-066 Cracow, Poland; Department of Molecular Biology, University of Gdańsk, Wita Stwosza 59, 80-308 Gdańsk, Poland

**Keywords:** *Wolbachia*, Apollo butterfly, Deformed wings, Reduced wings

## Abstract

Various insects contain maternally inherited endosymbiotic bacteria which can cause reproductive alterations, modulation of some physiological responses (like immunity, heat shock response, and oxidative stress response), and resistance to viral infections. In butterflies, *Wolbachia* sp. is the most frequent endosymbiont from this group, occurring in about 30 % of species tested to date. In this report, the presence of *Wolbachia*-specific DNA has been detected in apollo butterfly (*Parnassius apollo*). In the isolated population of this insect occurring in Pieniny National Park (Poland), malformed individuals with deformed or reduced wings appear with an exceptionally high frequency. Interestingly, while total DNA isolated from most (about 85 %) normal insects contained *Wolbachia*-specific sequences detected by PCR, such sequences were absent in a large fraction (70 %) of individuals with deformed wings and in all tested individuals with reduced wings. These results indicate for the first time the correlation between malformation of wings and the absence of *Wolbachia* sp. in insects. Although the lack of the endosymbiotic bacteria cannot be considered as the sole cause of the deformation or reduction of wings, one might suggest that *Wolbachia* sp. could play a protective role in the ontogenetic development of apollo butterfly.

## Introduction

The genus *Wolbachia* groups as a maternally inherited endosymbiotic α-proteobacteria. They live inside of cells of arthropods and filarial nematodes (Serbus et al. 2008). In insects, *Wolbachia* sp. can cause reproductive alterations, like feminization, parthenogenesis, male killing, and cytoplasmic incompatibility (Salunkhe et al. 2014). However these bacteria may also provide particular ecological benefits to the host (Saridaki and Bourtzis 2010). These mutualistic *Wolbachia*-host interactions include gene expression-dependent modulation of host immunity, heat shock response, and oxidative stress response, prevention of nurse cells apoptosis, and resultant effective maturation of oocytes, as well as host resistance to various viral infections (reviewed by Saridaki and Bourtzis 2010). Intriguingly, some effects of *Wolbachia* sp. on their hosts depend on the bacterial strain and the insect species (discussed by Iturbe-Ormaetxe and O’Neill 2007).

In butterflies (Lepidoptera), it was reported that about 30 % of species are infected with *Wolbachia* sp. (Russell et al. 2012). Interestingly, *Wolbachia* sp. is a highly predominant, heritable symbiont in these insects, as the presence of other such microorganisms (*Arsenophonus* spp., *Cardinium hertigii*, *Hamiltonella defensa*, and *Spiroplasma* spp.) was very rare in all tested families but Riodinidae (Russell et al. 2012). In Papilionidae, *Wolbachia* sp. was the only detected endosymbiont, and occurred in 20 % of tested species (Russell et al. 2012).

Apollo butterfly (*Parnassius apollo*) is a representative of Papilionidae. This butterfly was quite common in Europe in the nineteenth century. However, its distribution and population size have declined severely during the twentieth century, thus, it is near threatened now (Nakonieczny et al. 2007; van Swaay et al. 2010; Łozowski et al 2014). In this light, it is not a surprise that various programs focused on protection and survival of *P. apollo* have been established. They are, however, challenging as the remaining populations are sometimes extremely small. This was the case in the Pieniny National Park (Poland), where the apollo butterfly population had declined to about 20-30 individuals in the early 1990s (Witkowski and Adamski 1996; Witkowski et al. 1997). The restitution of this population has been successful, but since the late 1990s often appearance of insects with deformed or severely reduced wings has been noted (Adamski and Witkowski 1999). The mechanisms of these malformations remain to be elucidated. In this work, in order to determine if the presence of *Wolbachia* sp. might influence insect wing development, by using the applied genetics approach, we tested the presence of *Wolbachia*-specific DNA in samples derived from normal and malformed apollo butterflies.

## Materials and methods

### Insects

**Fig. 1:**
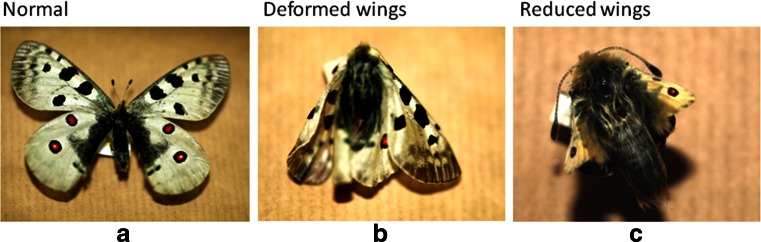
Examples of *P. apollo* individuals with normal (panel a), deformed (panel b), or reduced (panel c) wings. Photographs were made by the authors

All insects used in this work were from the collection of the Pieniny National Park (specimens were collected in years 1991–2007). The permission for the use of this material has been obtained from the Director of the Pieniny Natonal Park (permission no. PB-5232-24/07, topic ID: p0748). For experiments, five individuals of *Pieris rapae* (used as a positive control, reported previously to contain *Wolbachia* sp.; Tagami and Miura 2004), and 52 individuals of *P. apollo* were employed. Among the *P. apollo* specimens, 22 had normal wings, ten had deformed wings, and 20 had reduced wings. Photographs of examples of individuals from each group are shown in Fig. 1.

### DNA isolation and amplification

Total DNA was isolated from a material withdrawn from legs of investigated insects. The Sherlock AX Purification Kit (A&A Biotechnology, Gdynia, Poland) was used according to the manufacturer’s instruction.

DNA fragments, corresponding to particular tested genes, were amplified by PCR with the use of primers which are listed in Table 1. Amplified DNA was separated by agarose gel electrophoresis and analyzed according to Sambrook and Russell (2001).

**Table 1:** Primers used in PCR

Gene	Primers (forward and reverse)	Reference
*dpp*	5′ AGA GAA CGT GGC GAG ACA CTG 5′ GAG GAA AGT TGC GTA GGA ACG	Kapan et al. (2006)
*hh*	5′ AAG GAA AAA CTG AAT ACG CTG GC 5′ CGA GAC GCC CCA ACT TTC C	Kapan et al. (2006)
*ptc*	5′ CTC CGA AGA AGG TCT GCC GCA AG 5′ AAT TCG TGC TCG TCG TAT TTT C	Kapan et al. (2006)
16S rRNA	5′ GGA ACA CCA GTG GCG AAG GCG TCT 5′ CTG TGT GAA ACC CGG ACG AAC C	This work, designed on the basis of the 16S rRNA gene of *Wolbachia* sp., using the OLIGO 6.7 software (Molecular Biology Insights Inc., Colorado Springs, CO)

## Results and discussion

To analyze the presence of *Wolbachia*-specific DNA in *P. apollo* individuals, total DNA was isolated from samples of legs of either normal (20 butterflies) or malformed (32 butterflies) insects. In addition, for external positive control, DNA was isolated analogously from wild-type *Pieris rapae* (five butterflies), as the presence of *Wolbachia* sp. in this species has already been reported (Tagami and Miura 2004).

The quality of DNA templates were proved by PCR reactions with primers for amplification of fragments of *dpp*, *hh*, and *ptc* genes, present in the insect genome (Table 1). The product of amplification of the fragment of the 16S RNA gene of *Wolbachia* sp., obtained with the specific primers and using DNA isolated from wild-type *P. rapae*, was 336 bp long, as expected from the *Wolbachia* sp. genome analysis.

When the presence of the *Wolbachia*-specific DNA fragment after PCR with templates derived from *P. apollo* samples was tested, the 336 bp amplification product has been detected in the reaction containing the material from 19 out of 22 normal butterflies (Table 2). However, such a specific signal could be observed in only three out of ten individuals with deformed wings. Moreover, none of the 20 butterflies with reduced wings revealed the presence of *Wolbachia* sp. in this test (Table 2).

**Table 2:** Results of PCR-mediated DNA amplification with the use of indicated templates and primers specific to the 16S rRNA gene of *Wolbachia* sp

Species and characteristics	Number of individuals used for DNA isolation		
	All tested	With *Wolbachia* specific PCR product	Without *Wolbachia* specific PCR product
*P. rapae* (normal)	5	5	0
*P. apollo* (normal)	22	19	3
*P. apollo* (with deformed wings)	10	3	7
*P. apollo* (with reduced wings)	20	0	20

The results presented in Table 2 confirmed that *P. rapae* cells contain *Wolbachia* sp., and indicated that this endosymbiont is also present in *P. apollo*. Nevertheless, the most intriguing result is the strong correlation between wing malformations (deformation and reduction) and the absence of *Wolbachia* sp. To our knowledge, this is the first indication that this maternally inherited endosymbiotic bacterium might be important for proper development of wings in insects. Since *Wolbachia* sp. was found in only 20 % of tested species from Papilionacae, and it was absent in some normal individuals of *P. apollo*, while present in some butterflies with deformed wings (Table 2), a possibility that the lack of this bacterium might determine wing malformation is unlikely. Nevertheless, the strong association of the absence of *Wolbachia* sp. with deformation and reduction of wings in apollo butterflies may suggest that this endosymbiont could protect its host from some deleterious factors or agents affecting insect development.

In summary, this is the first report showing a correlation between wing malformation and the absence of *Wolbachia* sp. in *P. apollo*. Although the mechanism of this phenomenon remains to be elucidated, we speculate that *Wolbachia* sp. might play a protective role in the ontogenetic development of this insect. Therefore, the potential application of the results presented in this report is a possibility of the selection of *Wolbachia*-positive individuals (based on genetic tests) of *P. apollo* for further works on regeneration of populations of this butterfly, and its protection.
